# COVID-19 and Masking Disparities: Qualitative Analysis of Trust on the CDC’s Facebook Page

**DOI:** 10.3390/ijerph20126062

**Published:** 2023-06-06

**Authors:** Andrea Laurent-Simpson

**Affiliations:** Department of Sociology, Dedman College of Humanities and Sciences, Southern Methodist University, Dallas, TX 75205, USA; alaurentsimpson@smu.edu

**Keywords:** risk, institutional trust, COVID-19, emerging infectious disease, social media, masking

## Abstract

This exploratory paper examines individual levels of risk assessment as impacting institutional trust in the CDC while also contributing to disparities in expressed willingness to mask early in the COVID-19 pandemic. Using both content and thematic analysis of the CDC’s Facebook (FB) page from April 2020 and Gidden’s modern risk society theory, I consider how social media (SM) users retrospectively perceived a dramatic change in public health (PH) advisory—from the CDC advising against masking in February 2020 (Time 1) to advising the use of “do-it-yourself” (DIY) cloth masking in April 2020 (Time 2)—through a lens of prior, self-guided research. Expressed “knowledge” of masking as preventative (or not) yielded unwavering and sometimes increasing distrust in the CDC based on user perception of the “correct” advisory, regardless of the CDC’s position at Time 1 or Time 2. Simultaneously, disparities in masking behaviors appeared to be driven not by CDC guidance but by this same self-guided research. I show this via three themes: (1) claims of ineffectiveness for DIY masking (do not trust CDC now—no masking from the start); (2) conflict between the first and second CDC advisories on masking (do not trust CDC—either already masking anyway or will now); (3) disappointed in the CDC for length of time taken to make a DIY mask recommendation (do not trust CDC—either already masking anyway or will mask now). I discuss the imperative nature of two-way engagement with SM users by PH rather than using SM as a one-way mode of advisory dissemination. This and other recommendations may decrease disparities in preventative behaviors based on individual-level risk assessment as well as increase institutional trust and transparency.

## 1. Introduction

The original identification of SARS-CoV-2 in Wuhan, China, in December 2019 was followed by fast-paced, global spread of COVID-19. After initially deciding not to label SARS-CoV-2 a Public Health Emergency of International Concern in January 2020, the World Health Organization declared COVID-19 a pandemic in March 2020 [1]. In the United States, the first laboratory confirmed case was identified in Washington in January 2020, and new cases were increasingly discovered in the coming weeks. As the United States Food and Drug Administration began development and dissemination of emergency use tests in early February [1], the United States Centers for Disease Control and Prevention (CDC) was tasked as a public health authority (PHA) with providing prevention guidance to the public. Earliest measures included avoiding close contact with sick people; covering coughs and sneezes; keeping hands away from the face; and staying home when sick [2].

One of the CDC’s earliest prevention advisories regarding masking in the context of the pandemic appeared on Facebook (FB) on 27 February 2020 (Time 1), advising U.S. citizens against the use of face masks. The post noted that “only healthcare professionals caring for COVID-19 patients, people who are sick with COVID-19, or in some cases people caring for patients who are sick with COVID-19 need precautions like a facemask to help limit their risk of spreading COVID-19.” [3]. By early April (Time 2), emerging research indicated that individuals could be asymptomatic or pre-symptomatic, unknowingly transmitting the virus and dramatically increasing spread [4]. As a result, the CDC changed its guidance, recommending that all citizens wear cloth facemasks “in public settings where other social distancing measures are difficult to maintain (e.g., grocery stores and pharmacies) especially in areas of significant community-based transmission.” [5].

### 1.1. Theory

This research uses a modern risk society lens as a foundation for why differences in institutional trust develop, the context in which individual-level risk assessment emerges, and resulting variance in trust levels. Ulrich Beck has conceptualized risk society as characteristic of modern, post-industrialized societies [6]. Risk society arises in the context of rapidly advancing, modern technologies that usurp institutional ability to calculate risks of new innovations. Whereas members of more traditional societies can lean on local knowledge, religion, and observations of others’ habits for behavioral guidance, the modern risk society, by its very nature, forces members to rely upon expert systems of knowledge represented by scientists and other elites whom the average person will never meet [7]. 

As scientific knowledge is built, expert actors within these systems inevitably disagree on the accuracy of pieces of knowledge as well as how to mitigate the risk that may emerge from technological, and in this case, medical innovation. Anthony Giddens argues that “the fact that experts frequently disagree becomes a familiar terrain for almost everyone [and]… the claim to universal legitimacy of science becomes much more disputed than before.” [8]. A growing inability of science to predict risk (and subsequently develop solutions for society that protect against innovation-driven hazards) creates a social context in which hazardous conditions that emerge due to innovation cannot be effectively planned for in advance [6].

As a result, Giddens has argued that modern risk society emerges in which society’s members, once broadly trusting of expert systems, become increasingly aware of the manufactured risk unintentionally created by elite institutional authority [9]. As a result, the general public increasingly doubts expert systems to adequately assess and solve for risk [7]. Individual members feel themselves to be at greater risk as a result of both advancing technology as well as the inability of the institutional structures once trusted to mitigate risk to do so now [9]. Indeed, prior research shows that institutional trust by individuals in the U.S. medical profession, for example, has fallen from 73% in 1966 to 34% in 2012 [10]. 

Subsequently, and due to this loss of institutional trust, the general public begins to take the task of risk assessment into their own hands in the face of ever-progressing scientific innovation that also creates manmade risk [9]. Individual-level research on particular risks of concern emerges as a way to mitigate personal insecurity. The layperson, attempting to mitigate one’s own risk, begins “doing one’s own research”, including reading peer-reviewed scientific literature; watching and reading the news; or even reaching out to significant, in-group members for their thoughts on the issue to manage one’s own risk potential.

### 1.2. Summary and Grounded Theory Outcomes

**Figure 1:**
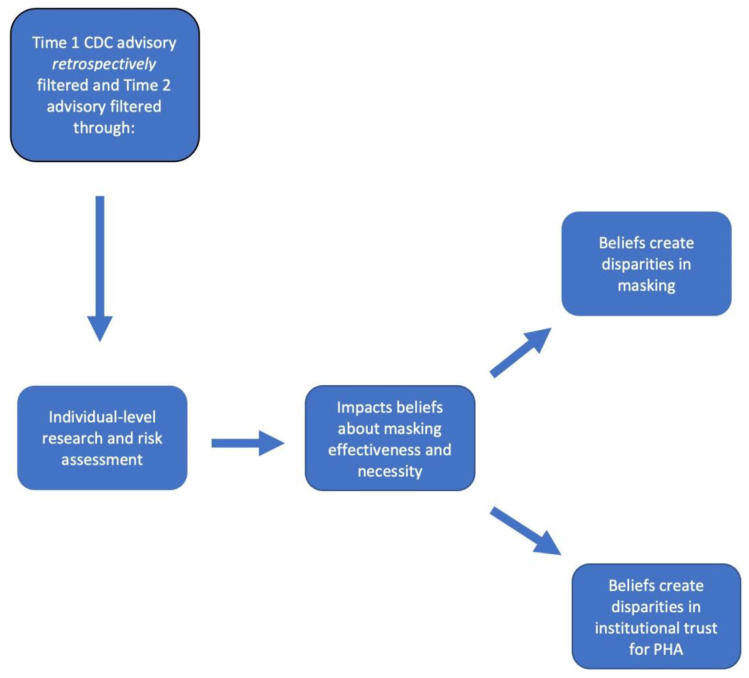
Theoretical argument.

In the context of the COVID-19 pandemic and CDC guidance regarding masking, I argue that CDC masking advisories were filtered through individual-level risk assessments already present for users when the CDC posted both the initial as well as the second advisory on the need to (or not to) mask. Figure 1 models the argument that the differences in these pre-existing, individually generated assessments of masking effectiveness created masking disparities. Simultaneously, these same risk assessments impacted levels of trust in the CDC’s ability to competently advise the public regarding masking. The perceived necessity for masks during the earliest period of the pandemic in the United States created fluid differences in levels of trust of both the CDC itself as well as its advisories regarding masking. The trustworthiness of the CDC’s advisory depended heavily upon whether the current guidance supported a user’s own individual-level assessments regarding the efficacy of masking. Ultimately, both masking behaviors and reported trust in the CDC’s advisories were filtered through users’ individual-level risk assessments rather than the recommendations made by the CDC.

This study has importance to the public health literature for several reasons. First of all, work examining how individual-level research on health influences both trust in PHAs as well as disparities in potential advisory uptake is underdeveloped in the literature [11,12]. Second, existing research examining the use of SM for health-related issues is scant [13,14]. However, we know that the Internet, and SM in particular, are increasingly used by PHAs as widespread dissemination sites of health information [15,16,17]. As a result, research that uses SM acknowledges these platforms as increasingly important sites of investigation.

Third, existing research on disparities in advisory uptake during COVID-19 and other emerging infectious diseases has focused on demographic variables to explain differences in preventive behavior [12]. These are valuable analyses with respect to the social determinants of health, to be sure. However, it is also important to consider how sociohistorical processes related to innovation risk and the resulting individual reflexivity required to assess risk is contributing to disparities in preventative behavior. Indeed, a large and growing body of literature has examined how self-reflexive research in the era of modern risk has created behavioral disparities and reinforced institutional distrust in many health contexts. This includes emerging infectious disease [11,18,19]; chronic disease [20]; contested illness [21]; and risk language in the media as reinforcing health risks [22]. Using a modern risk society lens to analyze why users on the CDC’s FB page felt the need to do their own risk assessments in the first place enables researchers to place these choices in sociohistorical contexts of ever-growing, historical distrust in social institutions.

### 1.3. Research Objectives and Questions

One of the objectives of this paper is theory generation that can be used in future quantitative work (see Section 2.3 below for more on this). This objective will lead to a better understanding of underlying, and oftentimes hidden, motivations (such as individual-level risk assessment) for the uptake of public health preventative measures (such as masking) that big data and computational and statistical designs might not otherwise pick up in analysis. A second research objective is to explore how individual levels of research on emerging infectious diseases like COVID-19 impact advisory uptake of preventative measures (e.g., masking) as well as how that same research impacts institutional trust.

These research objectives lead to the following questions in this paper: (1) Did individual-level research about COVID-19 and masking efficacy during the pandemic create masking disparities, as self-reported on the CDC’s FB page?; (2) How does individual-level research about COVID-19 and masking efficacy during the pandemic impact institutional trust, as expressed on the CDC’s FB page, in the CDC as a PHA?

## 2. Related Works

### 2.1. Institutional Trust and Rapid Change

As others have noted, prevention of disease spread in society is a fundamental function of PHAs like the CDC, and clear, consistent communication with the public about risk and prevention is key to stemming transmission [23,24,25]. However, compliance with PH advisories, especially those that require significant changes to daily routines like COVID-19 has, is dependent upon individual risk perception and trust in government, amongst other factors [24,25]. Because of the nature of COVID-19, governments and public health authorities have had to work at break-neck speed to make complex policy decisions in the context of nascent but rapidly evolving science [26]. Often, in the earliest days, these decisions were made, in better scenarios, with pre-print research, and in the worst scenarios, based on what PHAs did not know about the transmission of the virus [26]. As a result, PH guidance in the United States, especially regarding masking, risked the appearance of contradicting itself to the lay public, even while PH researchers were empirically building on recent scientific research. Public trust and cooperation is one of the many variables on which PHAs must depend when working to stem spread of an emerging infectious disease [25,27]. However, the appearance of backtracking on guidance is significant in the public eye and can be viewed as evidence supporting why PHAs should not be trusted. Inconsistent levels of trust from the public can, therefore, create disparities in preventative behavioral outcomes [25].

### 2.2. COVID-19, Masking, and SM

Extant research examining cross-cultural disparities in masking outcomes during the pandemic has highlighted a multitude of factors impacting this preventive behavior. This work includes the likelihood of masking during COVID-19 as associated with mainstream media or SM viewing [28]; pre-existing attitudes, social pressure, and perceived usefulness and benefits [29,30]; social norms and risk perceptions [30,31]; efficacy perception and prevalence within society [32]; and personalization of masks for identity expression [33].

Recent systematic social scientific research that has specifically explored perceptions of masking from around the world on social media outlets provides a more nuanced understanding of public, digitized debate regarding masking. Indicating the importance of masking discussion to the general public on SM, masking was one of two main prevention methods noted in English language tweets [34]. Further highlighting masking as a significant source of SM commentary, the 30 most frequently shared articles on FB regarding COVID-19 revealed that masking articles were the second most commonly shared articles, bested only by articles that discussed medication [35].

Research specific to increased understanding of how masking was perceived on SM during the pandemic is informative. One large, randomized sample taken from Twitter in June 2020 indicated that the platform was being used to create a community network of clusters with specific influencers, politicians, and general public persons present. User themes and hashtags appeared to center around encouragement to “mask up” [36]. Other research has utilized online experimentation in the U.S. to examine how masking misinformation rebutted by experts impacts user attitudes and perceptions regarding preventative behavior. While expert rebuttals were useful in increasing positive attitudes towards masking, subsequent disputes from SM users worked to create increased negative attitudes towards masking [37]. Still, other quantitative work has focused on public sentiment regarding masking and other preventative measures. Findings indicate that negative sentiment reduced when the current context of the pandemic became more severe (e.g., higher case counts) [38,39].

### 2.3. Current Methodology Used in SM Analysis of Masking

Most of the existing literature that considers SM, masking, and COVID-19 is classified as quantitative big data analysis that covers one-month time periods with samples ranging from 100,000 to over a million tweets [34,36,38,40]. Very little research exists that is qualitative in nature. More broadly, qualitative work examining the general public’s SM response to PH measures exists minimally and with broad thematic sweeps of attitudinal changes across the pandemic [41]. Other work considers healthcare professional response to PPE shortages with SM posts comprising one part of data collection [42]. To the author’s knowledge, qualitative work that focuses on SM response to masking as a preventative measure is scant at best. One paper examines tweets over a one-month period to show that pro- and anti-masking positions were both gendered in nature, with anti-masking tweets being more negative towards women’s uptake of the preventative measure [43].

Research on methodology may offer reasoning behind why so little qualitative work exists in the realm of SM research in general. Indeed, examining publicly generated content on SM platforms creates voluminous amounts of data. The sheer quantity of posts, regardless of whether the data collection site is FB, Twitter, Weibo, or any other site, lends itself towards fast-paced big data analysis that uses statistical workhorses such as Python and R for analysis. Likewise, researchers who use qualitative measures like thematic or content analysis that require manual coding of data are discouraged by the sheer volume of hundreds of thousands into the millions of data points across one month time periods [44]. However, while leaning on statistical analysis of such data is advantageous for testing hypotheses and predicting probabilities and outcomes, this approach is disadvantaged by the lack of in-depth theory generation that can inform the accurate building of quantitative designs and hypotheses via the self-reports of target populations [45].

As a result, this study uses qualitative content and thematic analysis of data taken from a one-day snapshot of SM posts (with a qualitatively “manageable” *n* = 1042 posts). Analysis examines how users of the CDC’s FB page retrospectively perceived the first CDC advisory on masking in the context of an at-the-time change in masking recommendations. Additionally, this paper examines how that change was filtered through users’ own risk assessment to impact disparities in reported masking intentions post-change in CDC advisory.

The topic of this paper, in particular, is an important area of research because while research conducted after the second advisory indicated that masking uptake in the U.S. occurred for 62% of the population days after and 76% one month following [46], FB data in this study suggest that choice to follow this preventive measure was moderated by complex patterns of trust in CDC advisories filtered through individual risk assessments. Differences in trust of government agencies and PHAs directed by individual-level risk assessments are an important driver of uptake for prevention strategies [11,12,47]. In this case, prior user-driven research (regarding both masking effectiveness and necessity) guided user-perceived accuracy of both of the CDC’s masking advisories. This same user-driven research also created disparities in reported masking outcomes on the CDC’s FB page. Examining self-reported levels of trust in PHA advisories is significant to scientific understanding of disparities in preventative behaviors such as masking. For example, a high level of institutional trust was a significant determinant of high levels of preventative behavior during the pandemic in Hubei, China [48]. Other research has shown that blame placed on public health authorities during earlier pandemics promotes distrust in PHAs, potentially eroding PH communication [49] and increasing behavioral disparities in guidance uptake.

## 3. Data and Methods

### 3.1. Methods and Data Source

This project asks two questions: (1) Did individual-level research about COVID-19 and masking efficacy during the pandemic create masking disparities, as self-reported on the CDC’s FB page?; (2) How does individual-level research about COVID-19 and masking efficacy during the pandemic impact institutional trust, as expressed on the CDC’s FB page, in the CDC as a PHA? The nature of both questions demanded a qualitative dataset that would provide substantive narratives highlighting differences in masking and institutional trust. As a result, I used a mixed methods approach for study design, using both conventional and directed content analysis as a means of analyzing data. Conventional content analysis (thematic) was appropriate for this study because it allows for inductive capture of themes in the data [50] that would not have been coded for if I had only been looking for confirmation of modern risk society. For example, locating trust as fluid in the data was one idea that came from this approach. Directed content analysis was also useful here as it allowed for grounding the project in pre-existing categories embedded in modern risk society, such as concrete trust in expert systems [51].

In the United States, the CDC uses multiple avenues of advisory dissemination, including television, radio, and SM contexts. I chose to examine SM data due to the inherent, two-way communication in which users can answer original posts, creating the potential for ongoing dialog regarding a variety of topics [11]. Furthermore, SM is widely used by laypeople as a platform for gathering health information [52]—especially as individuals increasingly seek to conduct their own health risk research—making SM platforms important sites of analysis. I chose the CDC’s FB page as the primary data collection site for this project because, in 2020, approximately 69% of American adults reported using FB regularly [53], with 54% of those users noting that they regularly looked for news on the platform [54]. Indeed, with the exception of YouTube, FB is by far the most widely used SM platform in the United States [53]. The CDC was chosen as the key public health authority because its mission, via the United States Public Health Service, is as the “main assessment and epidemiologic unit for the nation… serving the population as well as providing technical assistance to states and localities.” [55].

### 3.2. Data Collection

**Figure 2:**
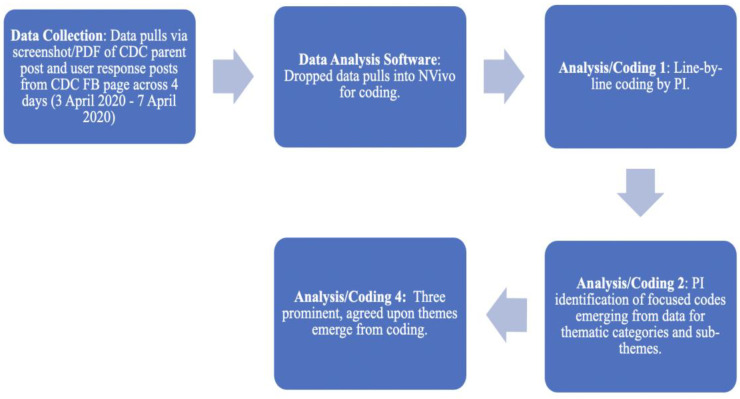
Content Analysis Flowchart.

As diagrammed in Figure 2, data were collected on the CDC FB page by using the internal FB search tool and the search terms “coronavirus” and (in March 2020) “COVID-19”. Especially regarding the search term “coronavirus”, the CDC parent post (PP) was then checked to ensure that it was specific to the novel coronavirus (and later COVID-19). Once the PP was verified as relevant, a screenshot and PDF were created for import into NVivo, a qualitative data analysis software program. PPs were pulled within 72 h from FB each time the CDC posted a new advisory or information. User response posts were also pulled in conjunction with each original CDC post. This process began on 28 January 2020, and continued until 10 April 2020, when lockdowns at my home institution made it difficult to continue gathering data. A total of 23 PPs were successfully mined in this manner. This paper focuses on a CDC PP made public on 3 April 2020, with user response posts in the dataset continuing until 7 April 2020. The PP was the CDC’s second masking advisory (Time 2) in which mask use for prevention changed from “not necessary except only under very unique circumstances” to “cloth masks should be used by everyone in public settings where social distancing measures are difficult to maintain”. Within this PP, 40 user-generated response posts (to the CDC) were made, with an additional 1001 response posts made in response to the 40 user-generated response posts. In total, 1042 posts comprised the data for this project. (For clarity, all user-generated posts, whether they are in direct response to the CDC or to other posters on the CDC FB page, will be referred to as posts or user posts). Users frequently referenced the CDC’s initial post (Time 1) in their response posts, and analysis in this project takes those retrospective accounts into consideration. Furthermore, user posts that are used as data points in this paper include a randomly-generated three letter alpha code as an identifier for each user.

### 3.3. Data Analysis

Grounded theory is a widely used qualitative approach that allows researchers to use participant narratives to inductively code data and generate themes [56]. The strength of this kind of analysis is that it offers depth of data that, while not generalizable or replicable in the way quantitative work might be, offers accuracy in analysis [45]. Major thematic content that arises from analysis of grounded theory research is useful in generating theories for future quantitative testing or other qualitative exploration [45]. Systematic line-by-line coding was used to search for substantive themes related to masking and attitudes towards the CDC. This process also allowed for inductive analysis that might reveal implicit meaning formation for users [57]. After identifying initial potential codes arising from the data, I then worked on focused coding—a process that culminated in combining less substantive codes into more powerful, substantive themes representative of the data [57]. This ultimately allowed the identification of three categories of trust in the second CDC advisory on masking aligned with resulting disparities in masking behaviors: (1) claims of ineffectiveness for DIY masking (do not trust CDC now—no masking from the start); (2) conflict between the first and second CDC advisories on masking (do not trust CDC—either already masking anyway or will now); (3) disappointed in the CDC for length of time taken to make a DIY mask recommendation (do not trust CDC—either already masking anyway or will mask now).

Data analysis of the sample of 1042 posts generated 42 initial coding categories with an additional 48 subcategories collapsed under various initial codes. Many of these initial codes did not generate thematic, focused codes (that is, very few pieces of data wound up coded under these categories) and thus were either ultimately abandoned in analysis or were later collapsed into other more precise focused codes that better represented the content of the user’s post. For example, one initial code, “Thanks to healthcare workers”, ultimately only garnered 6 pieces of data from the entire sample. This category was not deemed as collapsible into other codes while also having too few pieces of evidence to support it as a focused code. As a result, this initial code was abandoned. However, other initial codes, like “CDC lies”, were ultimately collapsed under a broader (and ultimately focused) code labelled “CDC not trustworthy”. As is typical of coding work in qualitative data analysis [56], sometimes a piece of data might be placed under multiple codes of analysis because the piece of data itself may be representative of more than just one code. In this study, 1042 posts generated a total of 1588 categorizations of data under various initial and, later, focused codes.

Regarding the themes in this paper, Table 1 depicts samples of data points that were categorized under each theme discussed in this paper. Table 1 also provides information on statements of trust (either direct or indirect statements regarding institutional trust in the CDC). Specific criteria were used to determine the inclusion of a piece of data as representative of focused codes. Data that were coded under Theme 1 had to meet at least one of the following parameters: (a) direct questioning of DIY mask as a viable preventative; (b) direct statements that masks do not work; (c) statements that advise, contrary to the Time 2 advisory, people not to mask. Data coded under Theme 2 had to meet at least one of the following parameters: (a) a reference to the Time 1 advisory as ill informed; (b) criticism of the CDC and/or government for not advising masks in Time 1 advisory; (c) direct or indirect statement about trust in the CDC related to conflicting advisories. Finally, data coded under Theme 3 were required to meet at least one of the following parameters: (a) indication that the CDC took too long to recommend DIY masking; (b) indication that individual masking outcomes would have been different if the CDC had told the general public earlier; (c) direct or indirect statement about trust in the CDC as related to length of time to Time 2 advisory. “Direct” or “indirect” statements about trust for each of these themes were determined by assessing if the data point outright noted distrust (direct—e.g., “How can you trust the CDC?”) or intimated distrust (indirect—e.g., I wouldn’t wear a mask unless I wanted to get sick”.) in some way.

## 4. Results

### 4.1. Theme 1—Dispute over Effectiveness of DIY Masking

How much a person fears something is shaped by what that person believes or thinks they know [58]. While social scientists debated why the general public should mask [59] or why they should not [26], user-generated content on the CDC’s FB page made it clear that posters had made up their minds. These positions were usually predicated on self-guided research and, sometimes, the “futility” regarding cloth masks. Indeed, FB posters made it clear that they had definitively made up their minds about the effectiveness of masking long before the CDC’s Time 2 advisory regarding masking during the pandemic.

Some users retrospectively noted that, while they had agreed with the Time 1 advisory discouraging mask use (save for very specific contexts), the second advisory guiding people to now use DIY cloth masks was inherently flawed. Arguments against the Time 2 advisory were predicated on the idea that DIY cloth masks were ineffective. Many of these posters self-reported that they had not been masking at all based on their own initial research regarding the dangers of COVID-19, with one user (User DGA) noting, “…If you thought you should wear a mask you did.”

A total of 124 user posts (12%) noted that cloth masks would not protect the wearer, with many citing their own research to that effect. For example, one post argued that:

…there’s no scientific evidence of the usefulness of universal masks, otherwise CDC would have issued a recommendation about it… Globally, scientific evidence about masks (surgical or N95 not to mention “cloth” masks) is sparse and makes it really hard for both the people and health organizations.

(User SLB)

Another poster (User RRC) noted with authority that cloth masks successfully protect the wearer “5% more or less depending on the extra steps you take to bulk it up. Maybe better than nothing but the problem is people are thinking they are safe with a cloth mask on. And they’re not”.

Other posters noted their own common sense as trumping the CDC’s Time 2 advisory. In response to one poster who was supportive of the all-around efficacy of DIY masks, a response post (User HAD) posited, “I see people with floppy masks on and say that is NOT protecting them from getting something. The edges let all sorts of ‘germs’ in. It is to stop the spread from the wearer, if they cough, sneeze, or spit while talking”. A user’s (User MDE) response to this post shot back, “How does that work? Your germs can’t get out but other people’s germs can get in? That doesn’t make sense. Masks aren’t one way”.

Posts coded under this theme appeared to have trusted the CDC’s Time 1 advisory in retrospect. As a result, evidence of individual risk assessment here also indicated devolving levels of institutional trust guided by posters’ evaluation of the CDC as now not trustworthy based on the Time 2 advisory. Indeed, one incredulous user (User YLF) marveled, “First we were told not to wear masks. Then, we were told to give up our masks. Now, wear cloth. Sorry—not trusting this [second advisory]”. Further, posts like this indicated a disparity in ever wearing masks for persons who saw the Time 2 as contradictory to self-derived knowledge.

Research that indicated cloth masks were useless also led 72 (7%) user posts to perceive the CDC as “lying”, displaying a full distrust of the Time 2 advisory. One user pointedly said:

The government didn’t want civilians buying up [N95] masks, adding to the shortage, needed by healthcare workers. So they were willing to sacrifice the general public. Also, the government is allowing the people to think BANDANAS work! They don’t! The virus is microscopic and goes right through regular fabric. I was a surgical nurse.

(User FPG)

### 4.2. Theme 2—Conflicting Mask Advisories

While 124 (12%) posts whose self-guided research supported the idea that cloth masks were not effective, 224 posts (21%) felt that cloth masks would be useful in protecting others from viral spread, with one post noting:

I hope everyone clearly understands that wearing masks is not to protect you from the virus but to protect others from you. Since you may have the virus without knowing it and if you cough and sneeze without covering yourself you will infect others without knowing it. So please wear masks everyone…

(User RFH)

Posts that indicated agreement with the Time 2 advisory might have elucidated an *evolving* trust in the PHA. Paradoxically, this was not necessarily the case. 74 (7%) posts were furious about the fact that the CDC had apparently contradicted itself, first advising masks for only the ill and their caretakers, and then, later, recommending that everyone wear cloth and/or DIY masks. These perceived conflicts in guidance were met with ire from those persons whose individual-level risk assessments had indicated a need to wear a mask from the beginning of the pandemic. For example, one user (User MRI) lamented, “It’s sad, but when the government said they [masks] weren’t necessary, red flags went up for me and I started wearing one in public. Now, you can’t purchase them anywhere.” For posters like this, risk assessment and research had led them to wear masks from the start, in opposition to the Time 1 advisory. Furthermore, it was clear that both of the CDC’s recommendations had been filtered through individual lenses of knowledge derived from their own research. Indeed, for these users, the Time 1 advisory had generated distrust in the CDC—and users had taken up the preventative tool anyway. Now, upon the Time 2 recommendation—one that agreed with these users—their distrust remained and, perhaps, even devolved some more. One irate user lamented:

They knew [face masks work]. If not, they SHOULD HAVE. Why did they not see China wearing masks???? Why did they not realize that all viruses are nonsymptomatic [sic] for days before symptoms appear???? All doctors and nurses and most people know this. Not waited til [sic] it was proven. IT was just common sense. CDC are murderers. No Excuses!

(User RFJ)

Another disgusted user added to the mix with:

How many people died before the CDC states the obvious. Started with: They don’t work. They work, but only for medical personnel, they work but only by stopping you from spreading it… you wouldn’t be able to wear one properly, they work but would make you overconfident. Did I miss any? Wear a fecking [sic] mask it’s obvious.

(User FSK)

Inductive analysis of posts like this brought to light that perceived conflict in advisories, especially for individual risk assessment that dictated masking from the start, retrospectively highlighted (Time 1) and reinforced (Time 2) a distrust in the CDC. This distrust was rooted in perceptions of risk built on research that not only included peer-reviewed, academic work but also on observations of N95 use by medical personnel, hospital policies, and even coaxing from employers.

Perception of risk related to not wearing a mask also appeared to be specifically related to observation of behaviors in Asian countries—especially of PH behaviors in these regions; analysis of financial markers in places including Hong Kong, Singapore, and Japan; as well as previous travel to various Asian locales that had historically worn masks. Indeed, as 51 (5%) posts highlighted these areas as important sources of information, it became clear that they were willing to trust various data flowing from Asia about masking in the pandemic before they would trust the Time 1 recommendation. While the CDC’s Time 2 recommendation was in line with pro-masking users’ own preventative behavior, the initial conflicting advisory coupled with the second yielded a devolution in institutional trust and accusations about mistakes that had been made by the CDC regarding the pandemic. Furthermore, and in line with the grounded theory in this paper, prior risk assessment that indicated the need for mask wearing created disparities in masking behavior (compared to those who agreed with the CDC’s initial advisory) as well as in institutional trust, with one user (User SLK) proud of the fact that they had bucked the CDC’s original advice: “I am glad that I’ve been making my own mask. It doesn’t have to be n95. Anything that can contain of protect the virus spreading is better than nothing”.

While advisory conflicts such as this were inevitable in the context of a novel pathogen that required rapidly evolving research and outcomes [60], such conflicts were frustrating for the general public. This was especially the case in the United States, a place where one angry poster proclaimed:

We can no longer trust the CDC… they are on Trump’s payroll. Cloth masks are NOT safe enough!!! Why is the richest and most powerful country in the world telling us how to do DIY life saving measures instead of providing n95 masks for all??

(User EAL)

Indeed, posters saw the CDC’s conflicting advisories as proof that an already brewing institutional distrust, coupled with posters’ own research, meant that the CDC could not be trusted [60].

### 4.3. Theme 3—CDC Waited Too Long

A third theme arose in this analysis in which 55 posts (5%) expressed frustration with the Time 2 advisory as well as an emergent distrust in the CDC as a PHA. That is, based on posted self-reports, these users felt that the CDC had waited too long to recommend any kind of masking. While not always apparent if these users had been masking all along, some posts made it clear that they had not been, based on Time 1 guidance, but that they would begin now. For example, one particularly accusatory post (User SCM) noted, “I’ve been sewing them [masks] like crazy for my family members. I happily would have started sewing them in January if the CDC had been more honest with the public about the necessity of wearing them”.

While posts like this indicated that these people would now begin using cloth masks, a sense of emergent betrayal and distrust also seemed apparent. For example, another poster (User NDN) responded, “CDC lied, people died. People could have been fashioning masks 2 months ago.” Another poster (User KKD) seemed to blame high testing numbers completely on the CDC while lamenting that people had not been wearing masks all along, saying, “If we would have all worn masks from the beginning the tested positive numbers would be lower. Blame the president all people want [but] this is solely on the CDC”.

In line with Gidden’s modern risk society lens [8,9], data also indicated that individuals had a responsibility to do their own research so that each person could build individual risk assessments, highlighting the fact that distrust in PHAs had been high from the start of the CDC’s Time 1 advisory not to mask. For those users who engaged in their own assessments, the CDC waiting too long to recommend DIY cloth masks did not matter to individual risk. One user matter-of-factly noted:

…just shows how everyone has duty to form [sic] own opinions and look out for themselves. I could not believe the things that were being said by “officials” when I have watched this unfold for months and new [sic] it was all fluff. It’s been airborne, it’s deadly, it leaves you with impaired lung function. Is it going to wipe out the entire world—no but a lot of people are at risk and warning public in January and taking steps would have made this a lot less deadly.

(User EVP)

For this poster, those who trusted macro-level expert system research (“officials”) to make individual-level masking decisions experienced a higher risk of death for themselves primarily because the CDC had waited too long to recommend cloth masking. Ultimately, retrospective narratives in this category supported the idea that disparities in masking behavior between Time 1 and Time 2 were rooted in higher levels of institutional trust at the Time 1 advisory (like those whose research did not support masking efficacy). However, a willingness to mask up in the face of the second advisory came alongside waning levels of institutional trust at Time 2.

## 5. Discussion

Regarding the first research question on the impact of individual-level research during the pandemic as yielding masking disparities, this research found that some posters did lean on their own research to make masking decisions. Indeed, 12% of users claimed that cloth and DIY masking, as recommended by the CDC, was ineffective. Another 7% noted that the CDC was lying about the effectiveness of cloth and DIY masks as a ruse to change the general public’s drive to acquire N95 masks. Individual-level research was thematic in these posts as a reason for ignoring the Time 2 advisory that people in the United States turn to masking as a preventative. Thus, disparities in masking arose for those users who leaned on their own research to justify the non-use of DIY and cloth masks.

The second research question asked if individual-level research regarding masking during the pandemic impacted institutional trust in the CDC. The second theme generated in this analysis made it clear that distrust in the CDC was sewn by what appeared to users to be conflicting advisories (Time 1 vs. Time 2). The third theme highlighted a general concern and subsequent distrust over how long the CDC took to finally issue a pro-masking advisory (Time 2). This research started from the position that anti-maskers, those individuals who had never had any intention of wearing masks based on their own research, would socially construct an argument of distrust in the CDC—especially once the CDC moved to recommend cloth face masks. However, inductive analysis of response posts shifted this stance. While FB data certainly supported the presence of an anti-masking contingent that did not believe in the effectiveness of DIY masking, it also laid bare that much of the distrust displayed by users arose in individuals who had been more than willing to wear a mask at Time 1 based on either their own assessments (“conflicting advisories” theme) or existent institutional trust (“waited too long” theme). Indeed, many posters who expressed distrust in the CDC noted that they had been masking all along based on their own research. Furthermore, many posters lamented that, based on their own research, the CDC’s incompetence was clear, given that the public had been exposed to images of persons wearing masks in China since January. For example, one person (User SCQ) noted, “Also in early February, Chinese people was [sic] recommended to cover their face in public if they have to go out of their home. I’m so disappointed, that the US government appears to even knew [sic] less than I did”.

What are the takeaways of this analysis? First, it is important for PHAs to consider that the individual-level risk assessment inherent in Gidden’s modern risk society is one that can create behavioral disparities and devolving levels of trust in and of itself. That is, the propensity for self-driven risk assessment at the individual level is a variable that should be examined alongside other social determinants such as gender, race and ethnicity, and socioeconomic status. In this research, users who leaned on their own research filtered both Time 1 and Time 2 recommendations through self-developed risk assessment lenses. These assessments then guided decisions about masking behaviors, ultimately revealing disparities in preventative behavior founded in how individual research framed the utility of masking. It is possible that creating audience-specific risk and crisis communication would mitigate the kind of behavioral disparities illuminated in this paper and that plagued American masking behaviors early in the pandemic [25].

PHAs might consider offering PH guidance based on a variety of different perspectives that acknowledge the capability of the general public to do its own “homework” regarding illness. This is especially the case when new scientific knowledge appears to contradict itself, leading to potential public confusion and an increased reliance on individual-level research [25]. I am in no way suggesting that laypersons who watch media coverage, peruse peer-reviewed work, and observe other regions’ illness response mechanisms are the equivalent of scientific expertise. However, PHAs must realize that a modern risk society inherently guides individuals to seek ontological security for themselves—and if people have become wary of trusting “expert systems” such as the CDC, especially when inevitable scientific conflicts occur, then they will “double down” on their own research to center themselves.

PHAs should not discount this element of self-education as an affront to public health expertise but rather as an opportunity to build on that self-education. For example, the CDC might offer a website in which multiple resources of knowledge, both pro- and con- advisory, are offered for public consumption. This would serve to not only validate self-derived knowledge and risk assessment in the public but also allow individuals to consider, in a non-defensive context, scientific perspectives that vary from their own assessments. This approach would increase PHA transparency (a guiding principle of trust in PHA [61]), acknowledge the potential for members of the modern risk society to research illness, and potentially further minimize disparities in preventative behaviors based on that research.

Second, and related to the above, prior research has noted that healthcare decisions have evolved into a partnership between physicians and patients who have increasingly been encouraged by pharmaceutical companies to do their own research regarding prevention, diagnosis, and treatment [62,63]. Responses to PHA advisories should be viewed in the same way. That is, as researchers work to understand disparities in preventative behaviors such as masking during the pandemic, viewing members of the general public as health consumers who have developed their own risk profiles and tolerances will be important in understanding how to minimize disparities in preventative behaviors. Furthermore, understanding how this “consumer” framework reflects the lens with which the public chooses to (or not to) engage in a recommended preventative behavior, regardless of PHA recommendations, will be an important tool in developing PH strategies that create public trust rather than erode it.

Reflective of the first conclusion above, PHAs should not ignore the power of self-guided research to create disparities in preventative behaviors. Instead, and like the modern physician–patient relationship, these entities should acknowledge a willingness to partner with the citizenry in ways that are not simply “people should partner with us (PHAs) by doing this”. Rather, the approach should be “we openly acknowledge and validate the general public’s growing ability and desire to make decisions based on self-guided research and we want to partner with you in that endeavor”. However, as one poster (User CKR) grumpily noted, “I’m actually tired of hearing the public doesn’t know how to use masks and increase [sic] the problem rather than decrease it. You know, we are not stupid. If you learned how to use them, we can too”. PHAs like the CDC that express a willingness to partner with the public stand to appear transparent and trusting of an increasingly educated population. These entities may also decrease disparities in preventative behavior uptake by recognizing modern society’s relationship to healthcare as consumer based.

Finally, it is important to note that, based on this research, it could be concluded that the CDC, as a PHA, was “damned if it did, damned if it did not” in relation to how posters perceived both the initial and second masking advisories. PHAs have increasingly used SM platforms for the dissemination of recommendations and advisories [15]. As such, agencies involved in public relations must understand the potential benefit to PH of two-way communication with the general public for answering questions, decreasing health behavior disparities, and building trust [11]. While the CDC did use town halls on Twitter as a way of answering questions about the pandemic, these communications were usually very controlled with only particular questions addressed live by experts. SM outlets such as FB offer unique two-way opportunities for PHA to have frequent conversations with far more laypersons who are suspicious or distrusting of the agency. Yet, out of 1042 posts, only one post was made by the CDC—the second advisory that people should begin using DIY cloth masks. Efforts to directly answer even some user-generated concerns might prove fiscally and temporally expensive in terms of manpower, and it has been noted that expert rebuttals can encourage more layperson disputes [37,47]. However, the use of social media as a two-way mode of communication between PHA and the public has been shown to be an effective mode of engagement for mobilizing society during public health emergencies [64]. Addressing concerns posted on SM platforms such as FB is important in decreasing uptake disparities as well as building trust in the general public.

This research is limited in a few ways. The first limitation is encompassed by FB as a data source. While it is true that 80% of adult SM users in the U.S. use SM to gather health information, the demographics of specific users on the FB platform is impossible to determine [65]. The only clearcut identifiers are user-generated FB handles and profile pictures that cannot be gauged for authenticity. Thus, any attempt to analyze this data for demographic purposes would be inaccurate at best. However, as with any disparity in health behaviors, variables such as gender, race and ethnicity, socioeconomic status, and religion are impactful in the formation of attitudes. Religion and political affiliation, for example, have been shown to influence levels of trust in scientific authority [66], and political affiliation may have impacted mask wearing in the United States from the start of the pandemic [59]. Other research has shown that trust in the government varies by race, with whites trusting the government but doubting its competence, while African Americans do not trust the government and doubt its motives [67]. Future research should work to identify how social variables impact the drive to engage in individual-level risk assessment and how resulting assessments can create disparities in both preventative behaviors as well as institutional trust.

Another limitation lies in the fact that CDC FB users who posted regarding the CDC’s second masking advisory appeared to submit multiple original and response posts. These more frequent posters were mixed with messages from far less frequent posters. Critics may argue that this concentration of posts from a small contingent of FB users skews the findings of this research. However, this is not unusual, given the nature of SM data in general. Indeed, van Mierlo has noted that 1% of SM users generate the vast majority of user-generated content, with a much smaller minority of posting coming from about 9% of users. The other 90% of users on SM platforms are “lurkers,” users who read posts but never generate any of their own content [68]. As with an inability to identify demographic information on users, this is a valid issue when analyzing levels of trust. For example, what are the differences between those users who choose to post condemnation of the CDC (or, alternatively, support for) and those who choose only to read. Future research should consider how these differences may impact the willingness to discuss health behavior outcomes in relationship to individual-level risk assessments and trust in PHAs.

Another limitation can be found in the cross-sectional nature of the data used. It should be reiterated that this exploratory qualitative research analyzed cross-sectional data taken from the Time 2 advisory. This work also analyzed Time 2 posts for retrospective responses to the Time 1 advisory. Based on the findings discussed in this paper, future mixed methods work, bolstered by theory generation taken from qualitative analysis like that in this paper, should explore a quantitatively-driven longitudinal design that allows for analysis of self-guided risk assessment and its impact on the evolution of both preventative behaviors and institutional trust.

It may also be noted that the limited time frame during which data was collected for this paper (4-day window from the CDC’s parent post to the last post made) may be a limitation. However, with the understanding that this work is exploratory in nature and specifically built for theory generation to be applied in future research, the small window of data collection may not be considered solely limiting. Rather, consideration of this data as a snapshot of responses, both current and retrospective, allows researchers to observe “in-the-moment” of complete advisory reversal, how prior risk assessments can immediately impact both behavioral outcomes as well as institutional trust. Furthermore, this shortened timeframe may be seen as a tradeoff for the accuracy in analysis of user narratives derived from qualitative content analysis. Indeed, this work provides a strength in the existing literature that is currently steeped in big data, quantitative analyses that, while potentially generalizable in nature, are not terribly accurate or precise with individual-level themes and narratives.

## 6. Conclusions

This research grows the knowledge that we have regarding how individual-level research regarding health advisories during the COVID-19 pandemic influenced both masking disparities as well as institutional trust in PHAs. As modern risk society posits, citizenry of modern societies initially places high levels of trust in the expert systems that help society progress forward. However, this progress does not come without peril. An inherent lack of expert ability to predict what risks may arise with technological innovation (and what the costs those risks may bring) yields an ever-growing distrust in the expert systems that the general public so readily trusted in the past [9].

In this research, users on the CDC FB page who posted regarding the CDC’s second masking advisory demonstrated self-developed risk assessments about mask usage that appear to have been in place prior to the CDC’s recommendation for DIY masking. Theory generation from this inductive analysis suggests that individual-level risk assessment is used as a lens through which PH advisories are filtered. As a result, this lens creates disparities in both masking outcomes as well as in levels of institutional trust. Ultimately, this work highlights the need for PH researchers and PHA to include in both future qualitative analysis as well as statistical modeling how individual-level research and subsequent risk assessments factor into health behavior outcomes.

## Figures and Tables

**Table 1:** Themes, sample data points, and N of posts for collected data.

Major Themes	Sample Data Points (by Theme and Trust)	N of Posts
Disparities in Masking and Institutional Trust:		
Dispute Over Effectiveness of DIY Masking (do not trust CDC now, no masking from the start)	“Make sure your [sic] not wearing a mask. They don’t protect you.”—User KNZ (Indirect distrust) “If COVID can in fact go right through material, then why on earth would anyone settle for a material ‘mask?’”—User PMY (Indirect distrust) “They need to address this. It’s ridiculous that the CDC does not understand micron sizes of fabric and the size of COVID19. They have to know. It [second advisory] has to be about alleviating fear.”—User TKX (Direct distrust) “Making masks[s] will not work […] just stay home now that works.” User MKW (Indirect distrust) “Unfortunately homemade masks and clothes likes scarfs [sic] don’t protect from particles going through…”—User AAV (Indirect distrust)	N = 124
Conflicting Mask Advisories (do not trust CDC—either already masking anyway or will now)	“CDC was irresponsible in telling the general public not to wear masks for the sole reason that they didn’t have any [masks]. They should have told people from the beginning to fashion a mask. Instead they shamed people into not wearing them even if they already had one they could have used.”—User CIU (Direct distrust) “Really CDC [sic], you are supposed to be the smartest people, hospitals follow your guidelines… Before airborne, now droplet. Before no mask now wear mask guidelines. No n95 when in fact that’s the best for now to protect the HC [healthcare] workers.”—User KGT (Indirect distrust) “The U.S. government knew all of this for months. They didn’t prepare and when they realized they were screwed because of inaction they tried to tell us not to wear masks to save them for the medical field. It NEVER made sense not to wear a mask.” User TCS (Direct distrust)	N = 74
Disappointment in CDC for Length of Time to Pro-Masking Advisory (do not trust CDC—either already masking anyway or will mask now)	“People could have been fashioning masks 2 months ago.”—User NDN (Indirect distrust) “They [CDC] have had this thing in a lab since Dec. Nothing new is coming out. They already know EVERYTHING about this virus. Trust me when I tell you, by the time they tell you to wear a mask, they already know it’s way worse than even they are telling you now.”—TCS (Direct distrust) “I’ve been sewing them [DIY masks] like crazy for my family members. I happily would have started sewing them in January if the CDC had been more honest with the public about the necessity of wearing them.”—User SCM (Direct distrust)	N = 55

## Data Availability

Data from this research are publicly available on FB and can be retrieved from https://www.facebook.com/cdc/posts/pfbid02nb49qW64gay2Ta7VSNvXd3yRuGe6QHAgggtqB2yoTjB7d1e6kivWGFYcWfVhendjl?comment_id=10157784682631026 (accessed on 9 January 2023).
